# Effects of Unilateral Transcranial Direct Current Stimulation of Left Prefrontal Cortex on Processing and Memory of Emotional Visual Stimuli

**DOI:** 10.1371/journal.pone.0159555

**Published:** 2016-07-19

**Authors:** Stefania Balzarotti, Barbara Colombo

**Affiliations:** 1 Department of Psychology, Catholic University of the Sacred Heart, Largo Gemelli 1, 20123 Milano, Italy; 2 Division of Education and Human Studies, Champlain College, 251 South Willard Street, Burlington, VT 05402, United States of America; University Medical Center Goettingen, GERMANY

## Abstract

The dorsolateral prefrontal cortex (DLPFC) is generally thought to be involved in affect and emotional processing; however, the specific contribution of each hemisphere continues to be debated. In the present study, we employed unilateral tDCS to test the unique contribution of left DLPFC in the encoding and retrieval of emotional stimuli in healthy subjects. Forty-two right handed undergraduate students received either anodal, cathodal or sham stimulation of left DLPFC while viewing neutral, pleasant, and unpleasant pictures. After completing a filler task, participants were asked to remember as many pictures as possible. Results showed that participants were able to remember a larger amount of emotional (both pleasant and unpleasant) pictures than of neutral ones, regardless of the type of tDCS condition. Participants who received anodal stimulation recalled a significantly higher number of pleasant images than participants in the sham and cathodal conditions, while no differences emerged in the recall of neutral and unpleasant pictures. We conclude that our results provide some support to the role of left prefrontal cortex in the encoding and retrieval of pleasant stimuli.

## Introduction

Besides the well-established role of subcortical structures in the limbic system such as the amygdala in the processing of affective information, other components of the emotional processing network such as the prefrontal cortex (PFC) have received growing attention [1–4]. For instance, recent studies have shown that dorsolateral prefrontal cortex (DLPFC)–which is traditionally thought to be involved in purely cognitive and executive functions (e.g., working memory) − also contributes to emotional processes such as emotional judgment (i.e., evaluations of affective valence [3]) and memory encoding of emotional stimuli [2, 5–6]. The specific contribution of each hemisphere, however, continues to be debated. A number of studies support the valence-specific hypothesis, providing evidence of anterior asymmetry in favor of left prefrontal regions for the processing of pleasant or approach-related emotional stimuli, and of right prefrontal areas for the processing of unpleasant or withdrawal-related ones [2–3, 6, 7–9, 10–11]. By contrast, other studies do not report evidence consistent with hemispheric asymmetry in PFC [12–15]. More evidence on the specific role of the left DLPC are still needed, and the present paper aims at focusing on this specific aspect.

The understanding of how the brain is organized to process emotional information has relevant clinical implications. Prefrontal brain regions, and in particular right and left DLPFC, have been a focus of research examining the brain mechanisms underlying clinical depression [16–18]. A number of studies have linked depression with reduced activity in (especially) left DLPFC, which may account for the so-called negative emotional bias, that is, the tendency of depressed individuals to show enhanced attention to and preferential memory for emotional negative information [18–22]. For instance, one study [23] found that, compared to healthy subjects, depressed patients showed left DLPFC hypoactivity while performing an emotional judgment task. Also, the less activity in left DLPFC, the less positively the patients evaluated positive emotional pictures.

Drawing on these findings, a number of attempts have been made to treat major depression by altering the balance between left and right prefrontal areas using brain stimulation techniques such as transcranial Direct Current Stimulation (tDCS) [24–26]. The tDCS is a non-invasive technique delivering weak electrical current through two electrodes (one anode and one cathode) positioned over the scalp, with cortical excitability increased under anodal and decreased under cathodal stimulation [27–28]. In neuropsychological research, tDCS is commonly employed for the rehabilitation of various disorders, as well as for the experimental study of cognitive processes in healthy subjects (for a review, see [29]). Thus far, however, the use of prefrontal tDCS in the treatment of depression has shown inconsistent results, with some studies finding a reduction of depressive symptoms after anodal stimulation of l-DLPFC [24, 30], and other studies showing no beneficial effects [31–32].

Few studies have so far examined the effects of prefrontal tDCS on emotional processing in healthy individuals [4, 33–37]. These studies measured the effects of brain stimulation on subjects’ performance on a range of cognitive tasks (e.g., valence ratings, recognition tasks, memory encoding) that require the processing of emotional information (either visual scenes or emotional facial expressions). Overall, results are mixed. Some studies [33, 34, 36] have reported that anodal tDCS of left DLPFC as compared to sham (baseline) tDCS lowers the perception of unpleasantness while viewing negative images, thus supporting the valence hypothesis. By contrast, other studies have observed that anodal tDCS of l-DLPFC (relative to baseline) has a small beneficial effect on recognition of emotional stimuli, regardless of valence [4].

Starting from this theoretical background, the present study follows the approach of two recent studies that have used similar experimental paradigms to examine whether stimulation of left prefrontal areas affect memory encoding and retrieval of emotional images. Penolazzi et al. [37] found that left anodal/right cathodal tDCS of frontal and temporal areas while viewing emotional images facilitated free memory recall of unpleasant images, while left cathodal/right anodal stimulation improved the recall of pleasant ones—notably, this effect is opposite to what would be expected based on the valence-specific hypothesis. By contrast, Morgan et al. [35] failed to find any significant effect of either anodal or cathodal tDCS of left DLPFC on the speed or accuracy of memory retrieval; yet, the study did not include a sham condition. These studies represent a first important step toward the study of the role of prefrontal areas in emotional processing with tDCS; however, both studies have used bilateral tDCS (i.e., stimulation of the left hemisphere is combined with antagonistic stimulation of homologue right areas), making thus difficult to establish the relative contribution of each hemisphere, which, as discussed above, is still controversial. In other words, results may be due to a combination of facilitation exerted by anodal stimulation in one hemisphere and interference exerted by cathodal stimulation in the contralateral hemisphere [37].

The present study adds to previous research by employing unilateral tDCS to test the unique contribution of left DLPFC in the encoding and retrieval of emotional stimuli in healthy subjects. Specifically, we aimed at verifying whether tDCS of left DLPFC while viewing neutral, pleasant and unpleasant images influences recall in a subsequent memory effect paradigm. According to the valence hypothesis (i.e., the two hemispheres are specialized in processing stimuli with opposite emotional valence), we hypothesized that anodal stimulation of left DLPFC would selectively enhance retrieval of pleasant images, while we expected to observe impairment in pleasant stimuli retrieval following left DLPFC cathodal stimulation.

## Materials and Method

### Participants

Forty-five right-handed undergraduate students from different disciplines (23 females; mean age = 21.55, SD = 1.17) joined the study. Participants were volunteers and received no credit or compensation for their participation. None of the participants had a history of neurological or psychiatric disorders. Due to technical problems during the stimulation, however, data of three participants were discarded from the analyses.

The study was conducted in accordance with the ethical standards of the Declaration of Helsinki and participants were free to withdraw from the study at any time. Participants provided written informed consent to the study by signing a printed consent form. The study and consent procedures received ethical approval from the Ethics Committee of the Faculty of Psychology of Catholic University in Milano. Although subjects were informed on the procedures, they were not aware of the goals of the study until after their participation had ended.

### Transcranial Direct Current Stimulation (tDCS) Protocol

The tDCS equipment used in the study (HDC Series by Newronika S.r.l, Milano) consisted of two sponge-based electrodes (25 cm^2^, 5cmx5cm). In order to constrain tDCS application to one hemisphere, one electrode (either the anodal or the cathodal one, according to the stimulation condition) was positioned on the subject’s scalp and the other on the ipsilateral mastoid. This specific montage has been used in previous studies [38], and its effectiveness discussed in a recent review [39]. Participants’ left DLPFC—identified through the 10–20 EEG international system (F3 electrode position)–was stimulated at a constant current of 1.5 mA for 15 minutes, resulting in a current density of 0.032 mA/cm^2^ (calculated by using the formula J = I/A, where J = current density in amperes/m^2^, I = current through a conductor, in amperes, and A = cross-sectional area of the conductor, m^2^). Previous experiments have shown that this stimulation duration induces cortical excitability shifts stable for at least 1 h after the end of tDCS (studies on M1: [40–41]) and it is specifically effective on the DLPFC, using the same size electrodes [42–43].

The experiment had a randomized, sham-controlled, parallel, single blind design. Although the stimulation protocol was single blind, the researcher who conducted data coding and analysis was not aware of the stimulation condition of the participants. Participants were randomly assigned to one of three conditions, depending on the type of stimulation: anodal (n = 14), cathodal (n = 14), and sham (n = 14). In the anodal condition, the anode electrode was positioned on F3 and the cathode electrode on the ipsilateral mastoid. In the cathodal condition the two electrodes were switched (cathode over F3, anode over ipsilateral mastoid). In sham or baseline condition, electrodes were placed in the same positions of anodal and cathodal conditions; stimulation of 1.5 mA was delivered for 30 seconds, which has been demonstrated to be unable to modulate cognitive functions, but is perceivable enough to give participants the impression of being stimulated [44]. Sham conditions (i.e., sham anodal and sham cathodal) were randomly varied and balanced within the sham group (i.e., half participants received sham anodal and the other half sham cathodal stimulation).

Stimulation was initiated five minutes before the beginning of the encoding phase (i.e., before pictures started to be displayed on the screen). During this interval (habituation phase), participants were asked to relax. Picture presentation lasted for 10 minutes and finished synchronically with the stimulation.

### Stimuli

Stimuli consisted of a set of 60 images (20 neutral, 20 pleasant and 20 unpleasant) derived from the International Affective Pictures System (IAPS; [45]). More in detail, the pictures were selected on the basis of the IAPS content categories [46]. Neutral images comprised images depicting everyday objects (e.g., a lamp, a light bulb, wicker baskets, etc.). Pleasant pictures depicted contents eliciting different positive emotions according to the functional evolutionary approach on positive emotion [47]: anticipatory enthusiasm (food), awe (landscapes), excitement (attractive men/women), nurturing and attachment love (kittens, puppies with their mother). In a similar way, negative images depicted contents eliciting different negative emotions such as fear (e.g., threatening animals), disgust (e.g. dirty toilets, insects), sadness (e.g., sad children), and anger (e.g., animals killed; [46]). Strong negative contents such as human mutilations were not considered. A description of the images employed in this study is provided in S1 Table.

The pictures were pre-tested on a sample of 40 undergraduate students (mean age = 21.70, SD = 2.29; 30 females). During the pre-test, the participants were individually asked to view (in random presentation order) and rate each picture for valence and arousal on the 9-point scale of the Self-Assessment Manikin [45]. Mean scores and standard deviations are shown in S1 Table. Pleasant and unpleasant pictures differed from neutral ones in terms of both valence (pleasant vs. neutral: *F*(1,39) = 514.38, *p* = .000; unpleasant vs. neutral: *F*(1,39) = 580.16, *p* = .000) and arousal (pleasant vs. neutral: *F*(1,39) = 369.39, *p* = .000; unpleasant vs. neutral: *F*(1,39) = 266.48, *p* = .000). Pleasant and unpleasant pictures differed from each other in terms of valence, *F*(1,39) = 834.10, *p* = .000, but elicited similar arousal levels, *F*(1,39) = 2.90, *p* = .10.

### Task and Procedure

Participants were seated with their eyes approximately 60 cm from a 15-inch monitor, and the stimuli were sized to fill the screen. Participants were instructed to look at images carefully for a subsequent recall test (intentional learning). The pictures were sequentially displayed in a random order for 10 seconds each, without any inter-stimulus interval. This encoding phase—during which tDCS was applied—was followed by a filler task that lasted 10 minutes (participants were asked to invent a story). The filler task was included in order to avoid active memory strategies during the retention interval. At the end of the filler task, participants were asked to remember as many images as possible (without following the presentation order) during a time interval of 10 minutes. In particular, participants were asked to write a short description for each image they could remember, in a way that, when reading this description, a person would be able to univocally identify the image among all the others. Three raters coded whether each picture was correctly recalled or not (1 = correct recall; 0 = not recalled) on the basis of the descriptions provided by the participants.

### Data Analysis

A Generalized Linear Mixed Model (GLMM) was run using SPSS 23. Logistic binary regression (logit) was selected as link function. tDCS Condition (anodal, cathodal, sham), picture Valence (pleasant, unpleasant, neutral), and their interaction were included in the model as fixed factors, while Participant and the type of Image were included as random variables to control for variance due to differences among participants and images. Recall (yes/no) was used as dichotomous dependent variable [48–49]. Bonferroni adjusted pairwise comparisons were employed to analyze significant effects.

## Results

**Fig 1:**
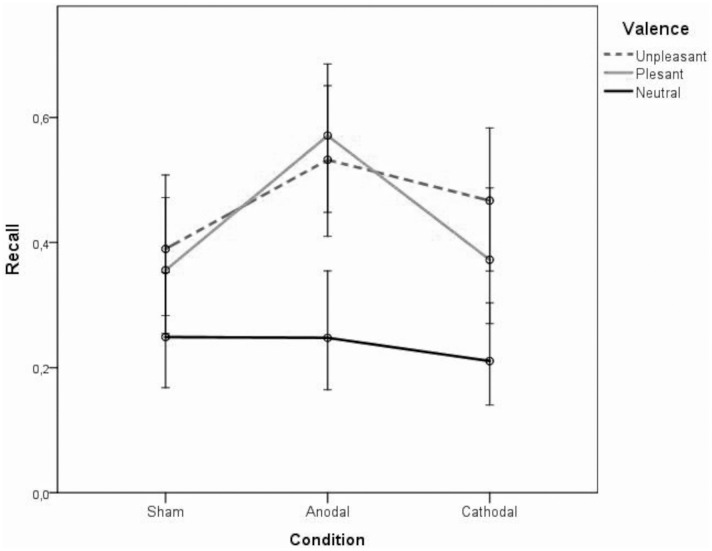
Task Performance. Mean proportions of correct recall as a function of picture valence (neutral, pleasant, unpleasant) and tDCS condition (anodal, cathodal, sham).

Results are shown in Fig 1. The analysis showed a significant main effect of Valence, *F*(2,2511) = 15.84, *p* = .000, with neutral images remembered to a less extent than pleasant and unpleasant ones. The interaction effect was also significant, *F*(4,2511) = 3.61, *p* = .006, while the main effect of condition was not significant, *F*(2,2511) = 1.82, *p* = .164. Estimates of fixed coefficients and random variance are reported in Tables 1 and 2. Adjusted pairwise comparisons (Table 3) showed that participants who received anodal tDCS were more likely to recall pleasant images than participants in the cathodal and sham conditions. By contrast, participants who received anodal tDCS were not more likely to recall unpleasant images than participants in the cathodal and sham conditions.

**Table 1:** Results of Generalized Linear Mixed Model: Fixed Coefficients.

Fixed Factor	β	SE	*t*	*p*	95% CI Lower limit	95% CI Upper limit
**Intercept**	-1.105	.253	-4.370	.000		
**Condition**						
**Cathodal**	-.217	.313	-.694	.488	-.831	-.397
**Anodal**	-.007	.324	-.021	.983	-.642	.628
**Valence**						
**Unpleasant**	.656	.250	2.629	.009	.167	1.145
**Pleasant**	.510	.250	2.041	.041	.020	1.001
**Condition x Valence**						
Cathodal * Unpleasant	.533	.265	2.010	.045	.013	1.053
Cathodal * Pleasant	.289	.266	1.086	.277	-.233	.812
Anodal * Unpleasant	.584	.269	2.173	.030	.054	1.111
Anodal * Pleasant	.886	.270	3.287	.001	.358	1.415

Note. Reference categories: Sham (Condition), Neutral (Valence).

**Table 2:** Results of Generalized Linear Mixed Model: Random Effects.

Fixed Factor	Variance	SE	*Z*	*p*	95% CI Lower limit	95% CI Upper limit
**Participant**	.436	.119	3.65	.000	.255	.746
**Image**	.271	.074	3.66	.000	.158	.462

**Table 3:** Generalized Liner Mixed Model: Bonferroni Adjusted Pairwise Comparisons.

Fixed Factor	β	SE	*t*	*p*	95% CI Lower limit	95% CI Upper limit
**Condition**						
**Cathodal vs. Sham**	.013	.059	.22	.829	-.103	.128
**Anodal vs. Sham**	.114	.065	1.76	.235	-.041	.269
**Anodal vs. Cathodal**	.101	.064	1.57	.235	-.044	.246
**Valence**						
**Unpleasant vs. Neutral**	.227	.043	5.26	.000	.124	.331
**Pleasant vs. Neutral**	.196	.043	4.56	.000	.100	.292
**Unpleasant vs. Pleasant**	.031	.048	.65	.515	-.063	.125
**Condition x Valence**						
**Unpleasant**						
**Cathodal vs. Sham**	.077	.073	1.07	.574	-.085	.240
**Anodal vs. Sham**	.142	.076	1.89	.179	-.039	.323
**Anodal vs. Cathodal**	.065	.076	.86	.574	-.096	.226
**Pleasant**						
**Cathodal vs. Sham**	.017	.069	.24	.809	-.119	.153
**Anodal vs. Sham**	.215	.074	2.91	.014	.038	.392
**Anodal vs. Cathodal**	.198	.073	2.70	.011	.034	.363
**Neutral**						
**Cathodal vs. Sham**	-.038	.055	-.69	1.00	-.171	.094
**Anodal vs. Sham**	-.001	.060	-.02	1.00	-.120	.118
**Anodal vs. Cathodal**	.037	.057	.65	1.00	-.090	.164
**Cathodal**						
**Unpleasant vs. Neutral**	.256	.054	4.77	.000	.128	.385
**Pleasant vs. Neutral**	.162	.051	3.15	.003	.047	.277
**Unpleasant vs. Pleasant**	.095	.058	1.64	.101	-.019	.208
**Anodal**						
**Unpleasant vs. Neutral**	.285	.056	5.05	.000	.158	.411
**Pleasant vs. Neutral**	.323	.056	5.80	.000	.190	.457
**Unpleasant vs. Pleasant**	-.039	.060	-.64	.520	-.157	.079
**Sham**						
**Unpleasant vs. Neutral**	.141	.054	2.62	.026	.012	.270
**Pleasant vs. Neutral**	.107	.053	2.03	.084	-.011	.225
**Unpleasant vs. Pleasant**	.034	.057	.60	.547	-.077	.145

## Discussion

In the present tDCS study, healthy volunteers received left DLPFC unilateral stimulation during presentation and encoding of neutral, pleasant and unpleasant pictures in order to test its effects on a subsequent free recall task. By using brain stimulation, our main goal was to examine the specific contribution of left DLPFC to the encoding and retrieval of emotional stimuli. So far, the few studies examining prefrontal tDCS effects on emotional processing and memory [35, 37] have in fact employed bilateral stimulation − which means that both left and right areas were simultaneously stimulated with opposite polarities − combining facilitation effects exerted by anodal stimulation in one hemisphere and interference effects exerted by cathodal stimulation in the contralateral hemisphere.

Our results revealed that participants were able to remember a larger amount of emotional (both pleasant and unpleasant) pictures than of neutral ones, regardless of the type of tDCS condition. This result is consistent with previous evidence showing that, in general, individuals remember more emotional events than non-emotional ones [50–52]. Also, left DLPFC stimulation influenced emotional memory as evidenced by the significant interaction between type of stimulation and picture valence. Specifically, participants in the anodal condition were significantly more likely to recall pleasant images than participants in the other conditions, thus supporting a facilitation effect of anodal tDCS of left DLPFC on the encoding and retrieval of pleasant stimuli. This result is consistent with the valence-specific hypothesis, which considers left prefrontal regions (including DLPFC) specialized in the processing of pleasant stimuli [7–10].

Our hypotheses, however, were only partially confirmed, as we did not find a corresponding interfering effect of cathodal stimulation of left DLPFC—participants in the cathodal and sham (i.e., absence of stimulation) conditions were able to recall a similar number of positive images. A recent meta-analytic review [53] has shown that if the anodal-excitation effect occurs quite commonly in cognitive studies (as measured by relevant cognitive tasks), the cathodal-inhibitory effect is far less stable. This failure of the cathode electrode to induce changes when applied over non-motor regions has been interpreted in different ways. First, it may reflect contralateral compensation processes given that cognitive functions are typically implemented in complex brain networks involving both hemispheres. Alternatively, it may be due to the fact that cognitive tasks highly activate brain regions during stimulation, so that cathodal stimulation acts in a ‘high competition’ environment [53].

Notably, no significant differences among conditions were found in the recall of unpleasant pictures. This result seems compatible with the valence hypothesis, which assumes right PFC as specialized in the processing of unpleasant stimuli. Since we employed left-unilateral rather than bilateral tDCS, stimulation of the left PFC (either anodal or cathodal) was not combined with antagonistic stimulation of homologue right areas.

Three limitations of this study bear noting. First, the size of the sample studied here did not allow testing for gender differences; however, prior research has observed that females generally remember more emotional stimuli than males [54–56]. Also, it has been shown that males and females may differ in the lateralization of emotional processing [55]. A second limitation is that the neurostimulation technique used in this study (i.e., tDCS)–though less invasive—has lower spatial resolution compared to other techniques, such as transcranial magnetic stimulation (TMS). Finally, in this study we employed an experimental paradigm used in prior research [37] targeting emotional retrieval and memory; for this reason, our results may not be generalized to other types of emotional processing. So far, research using brain stimulation in order to examine the contribution of prefrontal areas has employed a range of experimental tasks, including valence ratings, recognition tasks, memory encoding and retrieval of emotional stimuli—with stimulation generally applied during the presentation and encoding of stimuli (for an exception, see [35]). The mixed results obtained—with some studying supporting the valence hypothesis and other studies finding no support—suggest that valence effects may be dependent on the task used [10, 14], as well as on the type of cognitive process involved by the task itself (e.g., judgment, recognition, memory).

In conclusion, in this study, we found that unilateral anodal tDCS of left DLPFC during the encoding of emotional images facilitated participants’ subsequent recall of positive images. This result is relevant as it helps clarifying conflicting results reported by previous studies [35, 37] by focusing on the specific role of left DLPFC in emotional memory. Although further research using brain stimulation is needed to better evaluate the relative contribution of right prefrontal areas (as right DLPFC was not simulated in this study), our findings seem to support the role of left DLPFC in positive memory recall.

## Supporting Information

S1 File Main experiment data: Recall of neutral, positive, and negative pictures. (SAV)

S2 File Pre-test data A: Valence ratings. (SAV)

S3 File Pretest data B: Arousal ratings. (SAV)

S1 Table Pre-test: Means and standard deviations of valence and arousal ratings (Self-Assessment Manikin). (DOC)
