# Supplementary material for: Effects of Unilateral Transcranial Direct Current Stimulation of Left Prefrontal Cortex on Processing and Memory of Emotional Visual Stimuli
Source: PLoS One. 2016 Jul 19;11(7):e0159555. doi: 10.1371/journal.pone.0159555 (PMC4951131; doi:10.1371/journal.pone.0159555)
Supplement: S1 Table — (DOC) [file pone.0159555.s004.doc]

S1 Table. Pre-test: Means and standard deviations of valence and arousal ratings (Self-Assessment Manikin).

| **Picture** | | **Valence** | | **Arousal** | |
| --- | --- | --- | --- | --- | --- |
| **Code*** | **Description** | **M** | **SD** | **M** | **SD** |
| n_1 | Chairs | 4.76 | 0.70 | 1.98 | 1.66 |
| n_2 | Light bulb | 5.02 | 0.61 | 1.44 | 1.11 |
| n_3 | Wicker baskets | 5.02 | 0.61 | 1.54 | 1.01 |
| n_4 (7055) | Light bulb | 4.95 | 0.67 | 1.38 | 0.74 |
| n_5 | Glass | 4.93 | 0.57 | 1.44 | 1.13 |
| n_6 | Pen | 4.76 | 0.73 | 1.54 | 1.11 |
| n_7 | Windows | 5.32 | 0.79 | 1.88 | 1.52 |
| n_8 | Wheel | 4.68 | 0.76 | 1.24 | 0.74 |
| n_9 | Fork | 5.12 | 0.81 | 1.66 | 1.42 |
| n_10 | Buttons | 5.32 | 0.79 | 2.29 | 1.70 |
| n_11 | Lamp | 5.02 | 0.61 | 2.32 | 1.73 |
| n_12 | Street lamp | 5.24 | 0.70 | 1.98 | 1.66 |
| n_13 | Watering can | 4.85 | 0.74 | 1.48 | 1.21 |
| n_14 | Jars | 4.95 | 0.59 | 2.29 | 1.70 |
| n_15 (7150) | Umbrella | 4.85 | 0.79 | 1.27 | 0.82 |
| n_16 (7059) | Keyring | 4.93 | 0.41 | 1.39 | 0.84 |
| n_17 (7041) | Baskets | 5.15 | 0.57 | 1.38 | 0.81 |
| n_18 | Teapots | 4.93 | 0.52 | 1.46 | 0.88 |
| n_19 | Japanese teapot | 5.05 | 0.63 | 1.68 | 1.11 |
| n_20 | Tissues | 4.93 | 0.96 | 1.78 | .83 |
| **Total Neutral** | | **5.01** | **0.53** | **1.64** | **0.84** |
| p_1 | Tiramisu cake | 7.39 | 1.38 | 5.98 | 2.07 |
| p_2 | Focaccia bread | 7.20 | 1.29 | 5.83 | 2.07 |
| p_3 | Cake | 7.05 | 1.64 | 5.57 | 2.09 |
| p_4 | Pizza | 7.61 | 1.20 | 6.60 | 1.82 |
| p_5 | Dolphins (puppy-mom) | 7.68 | 1.11 | 5.75 | 2.35 |
| p_6 | Seals(puppy-mom) | 7.37 | 1.18 | 5.43 | 2.17 |
| p_7 | Bears(puppy-mom) | 7.61 | 1.24 | 5.70 | 2.08 |
| p_8 | Flowers | 6.76 | 0.86 | 3.93 | 1.80 |
| p_9 | Attractive man/woman | 7.15 | 1.26 | 6.02 | 2.09 |
| p_10 | Attractive man/woman | 7.51 | 1.25 | 5.76 | 2.42 |
| p_11 (4542) | Attractive man/woman | 6.63 | 1.11 | 4.65 | 1.81 |
| p_12 | Landscape nature | 7.93 | 0.96 | 5.85 | 2.12 |
| p_13 | Landscape nature | 7.56 | 1.03 | 5.60 | 2.19 |
| p_14 | Landscape nature | 7.68 | 1.15 | 6.05 | 1.66 |
| p_15 | Landscape city | 6.54 | 1.23 | 4.50 | 1.82 |
| p_16 | Landscape city | 6.95 | 1.16 | 4.92 | 2.04 |
| p_17 | Landscape nature | 7.51 | 1.25 | 5.77 | 2.07 |
| p_18 | Kittens | 7.24 | 1.85 | 5.68 | 2.20 |
| p_19 | Kittens | 7.83 | 1.18 | 5.85 | 2.26 |
| p_20 | Kittens | 7.54 | 1.05 | 5.80 | 2.15 |
| **Total Pleasant** | | **7.34** | **1.10** | **5.56** | **1.32** |
| neg_1 | Spider | 1.88 | 1.25 | 6.05 | 2.57 |
| neg_2 | Bed bug | 2.59 | 1.38 | 5.25 | 2.65 |
| neg_3 | Bugs | 1.58 | 0.90 | 6.18 | 2.19 |
| neg_4 | Shark | 3.05 | 1.88 | 5.50 | 2.11 |
| neg_5 (1026) | Snake | 3.05 | 1.62 | 5.33 | 2.27 |
| neg_6(9589) | Dental exam | 2.61 | 1.39 | 4.97 | 2.33 |
| neg_7 | Medical procedure | 2.10 | 1.26 | 5.87 | 2.32 |
| neg_8 | Dead hedgehog | 1.95 | 1.07 | 6.42 | 1.95 |
| neg_9 | Wound | 3.02 | 1.47 | 6.27 | 2.05 |
| neg_10 | Dirty latrine | 1.76 | 0.83 | 5.68 | 2.32 |
| neg_11 | Dead elephant | 2.34 | 1.32 | 5.58 | 2.35 |
| neg_12 | Ill-treated dog | 2.02 | 0.99 | 5.45 | 2.42 |
| neg_13 | Dog in a cage | 2.34 | 1.32 | 5.83 | 2.46 |
| neg_14 | Ill-treated dog | 3.88 | 1.73 | 6.40 | 2.09 |
| neg_15 | Sad begging child | 2.59 | 1.64 | 6.30 | 2.10 |
| neg_16 | Sad child | 2.68 | 1.47 | 5.90 | 2.11 |
| neg_17 | Poor African family | 3.68 | 1.94 | 5.05 | 2.25 |
| neg_18 (2688) | Bear hunted | 2.59 | 1.66 | 5.80 | 2.29 |
| neg_19 (2053) | Baby in incubator | 2.39 | 1.34 | 6.27 | 2.11 |
| neg_20 | Corpses | 1.49 | 0.95 | 7.71 | 1.60 |
| **Total Unpleasant** | | **2.46** | **1.36** | **5.87** | **1.48** |

*Note. When an original IAPS image was use, the number is reported between brackets.
